# Research on Spent LiFePO_4_ Electric Vehicle Battery Disposal and Its Life Cycle Inventory Collection in China

**DOI:** 10.3390/ijerph17238828

**Published:** 2020-11-27

**Authors:** Lingyun Zhu, Ming Chen

**Affiliations:** School of Mechanical Engineering, Shanghai Jiao Tong University, Shanghai 200240, China; woaimm129@sjtu.edu.cn

**Keywords:** LiFePO_4_, recycling, LCI, electric vehicles, dismantling, pyrolysis, lithium ion battery

## Abstract

The main research direction for the disposal of spent lithium-ion batteries is focused on the recovery of precious metals. However, few studies exist on the recycling of LiFePO_4_ electric vehicle (EV) batteries because of their low recycling value. In addition, a detailed life cycle inventory (LCI) of waste plays a significant role in its life cycle assessment (LCA) for an environmental perspective. In this study, an end-of-life (EOL) LiFePO_4_ EV battery is disposed to achieve the LCI result. The approach comprises manual dismantling of the battery pack/module and crushing and pyrolysis of cells. The authors classify the dismantling results and use different disposal methods, such as recycling or incineration. Regarding the environmental emissions during pyrolysis, the authors record and evaluate the results according to the experimental data, the bill of materials (BOM), the mass conservation, and the chemical reaction equations. In addition, the electricity power demand is related to the electricity mix in China, and the waste gases and solid residue are treated by using neutralization and landfill, respectively. Finally, the authors integrate the LCI data with analysis data and a background database (Ecoinvent). After the integration of the total emission and consumption data, the authors obtained the total detailed LCI resulting from the disposal of the LiFePO_4_ vehicle battery. This LCI mainly includes the consumption of energy and materials, and emissions to air, water, and soil, which can provide the basis for the future LCA of LiFePO_4_ (LFP) batteries. Furthermore, the potential of industrial scale process research on the disposal of spent LiFePO_4_ batteries is discussed.

## 1. Introduction

### 1.1. The Issue of End-of-Life Vehicle Battery

In recent years, emphasis on environmental protection has expanded the global market for electric vehicles (EVs). Moreover, many automobile manufacturers have developed their own EVs, such as the Leaf (Nissan), Prius (Toyota), Volt (Chevrolet), and Model S (Tesla). In China, the electric vehicle market is developing rapidly. In 2015, 331,092 electric vehicles were sold in China, making it the largest market in the world, compared to sales of only 8159 in 2011 [1]. Subsequently, China’s electric vehicle sales have occupied a dominant position [2]. In particular, vehicle manufacturers such as the Shanghai Automotive Industry Corporation (SAIC), BYD Auto Co. Ltd. (BYD, Shenzhen, China), and FAW Group Corporation (FAW, Changchun, China) have launched their own EVs.

However, such development will result in spent vehicle batteries. The electrodes of these batteries may contain heavy metals, for example, cobalt in LiCoO_2_ (LCO) batteries, nickel in Ni-MH batteries, and manganese in LiMn_2_O_4_ (LMO). Each of these heavy metals may be present in nickel cobalt manganese lithium (NCM) batteries. Heavy metals can pollute the soil, water, and air if not appropriately treated. LiFePO_4_ (LFP) batteries do not contain heavy metals, and their solvents comprise harmless (non-toxic) carbonate mixtures, additives, etc. However, lithium hexafluorophosphate (LiPF_6_), which is often used for lithium salt, is highly toxic. In addition, the decomposition of LiPF_6_ products results in hydrogen fluoride (HF), which is extremely harmful to human health [3]. An addition safety issue relates to the combustion of organic solvents, carbonate mixtures of ethylene carbonate (EC), dimethyl carbonate (DMC), ethyl methyl carbonate (EMC), propylene carbonate (PC), etc., which is a potential safety hazard due to the generation of unburned hydrocarbons and related oxygenated compounds [4].

In the life cycle concept, the disposal phase is an essential part of a product’s life. The life cycle relates to a product’s environmental impacts and economic benefits. Numerous developments and measures have been incorporated in laws, recycling technologies, and life cycle assessment (LCA) studies.

A large number of regions and countries have issued laws and regulations related to battery recycling [5]. The European Union enacted the directive 2006/66/EC [6,7], which covers all types of batteries and accumulators. In China, the government announced regulations and standards for used battery disposal. The Technical Policy for the Recovery of Automobile Products states that EV manufacturers should be responsible for the recycling and treatment of sold EV batteries [8]. The Circular Economy Promotion Law of the People’s Republic of China regulates the recycling of waste products, such as batteries, which are included in the mandatory recycling list [9]. In 2012, the Chinese government advocated the establishment of cascade utilization and recycling management systems for EV batteries [10]. The instructions of the “Vehicle Battery Industry Standard Conditions” stipulate that battery production enterprises must (i) meet the requirements of the environmental and occupational health and safety systems; (ii) recycle or treat waste created during the manufacturing phase; and (iii) engage with the vehicle manufacturer for the disposal procedure of used vehicle batteries [11]. “The Industry Standard Conditions of New Energy Vehicle Used Battery Utilization and Interim Administrative Measures” regulates the relevant recycling and disposal technologies and their efficiency requirements, and the distribution of recycling and disposal responsibilities among the stakeholders [12].

Globally, research into battery recycling has expanded rapidly. Currently, hydrometallurgical and pyrometallurgical processes are widely used to recycle spent lithium-ion batteries [13].

Pretreatment processes, which are usually a necessary preparatory step, may consist of electrochemical, mechanical (dismantling and crushing), and heat treatments, and physical separation. The pyrometallurgical process can remove the organic binder by heating. Moreover, it may use oxidization, reduction, and decomposition to attain metals and their oxidation compounds. The hydrometallurgy process is often divided into two main stages: leaching and extraction. Chemical leaching often dissolves the cathode material in lithium-ion batteries with acid. Extraction usually separates optional target products from the solution using special solvents. Studies on the leaching process primarily focus on leaching efficiency and influential factors, including the type and concentration of acid, temperature control, reaction time control, and the solid–liquid ratio [14,15,16,17,18,19,20,21]. In general, the main recycling target is valuable metals [22], such as cobalt and nickel in the forms of Co_2_O_3_, CoC_2_O_4_, and Ni(OH)_2_.

In addition, many studies have been conducted on the LCA of the environmental impact of EVs and batteries. These studies have analyzed energy consumption and emissions of different EV and battery technologies to achieve methods of sustainable improvement. Fisher et al. conducted an LCA of waste portable batteries with combinations of three collection scenarios and three recycling scenarios. They then analyzed the environmental benefit and financial cost of disposal [23]. Ellingsen et al. compiled an inventory for a lithium-ion nickel-cobalt-manganese traction battery and analyzed the impacts in the cradle-to-gate phase [24]. Notter et al. compiled a detailed life cycle inventory of the LiMn_2_O_4_ battery and compared the emissions and impacts between battery electric vehicles (BEV) and internal combustion engine vehicles (ICEV) [25]. Majeau-Bettez et al. conducted an LCA of three types of batteries, namely Ni-MH, NCM, and LFP batteries for plug-in hybrid vehicles and EVs [26]. However, the authors excluded an end-of-life disposal scenario from the inventory. Zackrisson et al. conducted the LCA of LFP batteries using different solvents during cell manufacturing to examine and optimize the design of batteries [27]. However, the recycling and disposal phase of spent batteries was simplified by a 500 km transportation route. Hendrickson et al. conducted the LCA of vehicle batteries, including LFP batteries, in California. In the compilation of the inventory for battery recycling, the authors made use of the background database built-in the GREET2 model [28].

### 1.2. Purpose

At present, the LFP battery plays an important role in the Chinese EV battery market (32% shares in 2019, China). Regarding the cell, the decomposition of LiPF_6_ can product toxic gases, and organic solvents also represent a safety issue. In addition, regulations and laws have implemented and targeted numerous measures and requirements for spent vehicle battery recycling and disposal. Consequently, it can be concluded that spent battery disposal is an important issue in China and should not be ignored.

However, the primary objective of battery recycling is the recovery of the valuable metals (Co, Ni, Ti, Cd, Cu) included in Ni-Cd, Ni-MH, and some kinds of Li-ion batteries [22]. This has resulted in a low level of interest in LFP recycling research. Nonetheless, to date, the main research focus of the life cycle assessment of EVs and their batteries is often the material extraction, manufacturing, and use phases. The maturation of the recycling industry is not yet at a high level, and few detailed studies have been conducted on the life cycle inventory (LCI) of LFP batteries during the recycling and disposal phases. As a result, simplified methods have been adopted in the industry, resulting in problems of accuracy. Hence, the authors of the current study recognize that research on the LCI of LFP battery disposal is necessary. The LCI case study of the LFP battery disposal phase will not only be a basic requirement of future LCAs but can also reflect the current disposal technology level and energy consumption status in China.

## 2. Method

### 2.1. Materials, the Total Disposal, and Inventory Program

**Figure 1:**
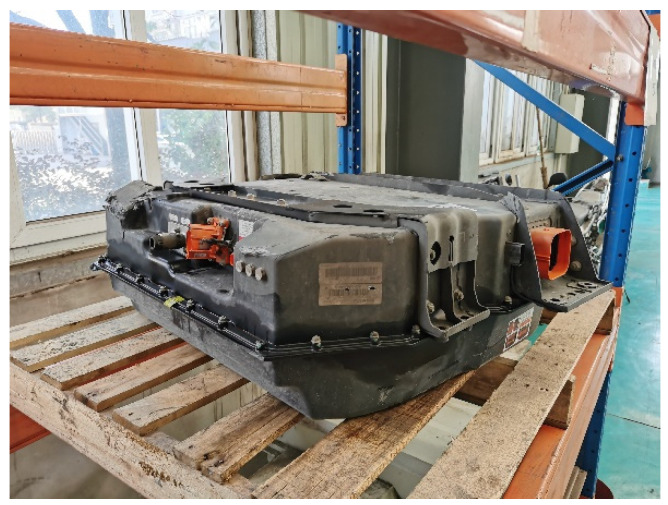
LiFePO_4_ electric vehicle (EV) battery pack.

The authors analyzed a type of LiFePO_4_ traction battery pack that is used in the Roewe 550 plug-in hybrid electric vehicle produced by SAIC (Figure 1). The battery pack consists of modules, cases, battery management systems (BMSs), and other related components. The basic technical details of the battery pack and cells are shown in Table 1. The cell is mainly composed of the shell, cathode and anode electrodes, separator, and electrolyte. The electrolyte consists of LiPF_6_ dissolved in organic solvents. In addition, the bill of materials (BOM) of the cell is shown in Table 2.

The recycling of vehicle batteries often starts with pretreatment, which mainly includes disassembly of the battery pack from the electric vehicle, the dismantling of the battery pack and modules, battery discharge, and other safety treatments [13,20]. For the treatment of batteries, as shown in the introduction, a large number of studies have mainly focused on the recovery of valuable metals by pyrometallurgy and hydrometallurgy methods. LFP cells have a low recycling value, but they also contain organic solvents with low flash and LiPF_6_, which can generate fluoride to air. Therefore, safety disposal for LFP cells can be given priority. In the authors’ previous research [13], the pyrolysis treatment method for nickel cobalt manganese lithium (NCM) batteries, which also contain organic solvents and LiPF_6_ similar to LFP batteries, has achieved a considerable treatment effect. Therefore, in this study, the authors make use of the pyrolysis method to treat the cells and employ the landfill disposal for the pyrolysis products.

**Figure 2:**
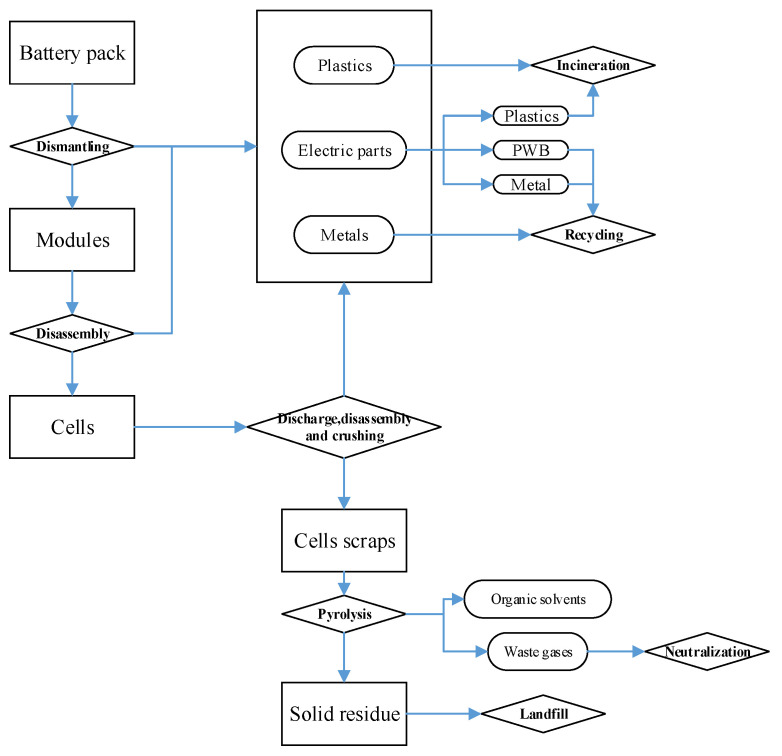
The total disposal process of LiFePO_4_ vehicle battery.

The process for the disposal of spent batteries is shown in Figure 2. In general, the authors adopted manual dismantling for the battery pack and module separation. Regarding cells’ disposal, the authors employed the discharging, crushing, and pyrolysis methods. The authors classified the dismantling results (without the internal material of the cells) and used different disposal methods, such as recycling or incineration for the internal material of the cells. After the dismantling and crushing phases, the cells’ internal materials were crushed to a size of 5–10 mm to prepare for the pyrolysis phase. Regarding the safe disposal of cells’ internal materials, the authors conducted the pyrolysis process in a furnace (rated power: 2.5 kW), and the cell scraps were thermally treated at 400 °C for 0.5 h per cell. Then, the emission gases (HF, POF_3_, and PF_5_) were neutralized by limewater and the pyrolysis solid residue was transported to landfill. The relevant key chemical reaction equations are shown below. Finally, the pyrolysis solid residue without much recycling value is treated at the landfill.

(1) $$LiPF_{6}\overset{}{\to }LiF+PF_{5}↑$$

(2) $$PF_{5}+H_{2}O\;\to \;2HF↑+POF_{3}↑$$

(3) $$POF_{3}+3H_{2}O\;\overset{}{\to }\;H_{3}PO_{4}+3HF↑$$

(4) $$2H_{3}PO_{4}+3Ca{\left(OH\right)}_{2}\;\overset{}{\to }\;Ca_{3}{\left(PO_{4}\right)}_{2}↓+6H_{2}O$$

(5) $$2HF+Ca{\left(OH\right)}_{2}\;\overset{}{\to }\;CaF_{2}↓+2H_{2}O↑$$

Regarding the inventory collection and calculation, the LCI data consist of foreground and background data, which include the energy and material consumption and emissions from the disposal processes of dismantling and crushing, the electricity power demand, and the treatment of the pyrolysis phase and its residue.

Regarding the foreground data, the authors recorded the amount of metals and other parts from the dismantling and crushing steps, the electricity demand of the equipment during pyrolysis, and the amount of pyrolysis residue. According to the mass conservation analysis of the bill of materials and the chemical reaction equations (Equations (1)–(5)), the authors calculated and evaluated the material consumption of CaO and nitrogen, the emissions of solvents to air, and emissions of Ca_3_(PO_4_)_2_ and CaF_2_ to solid during the pyrolysis process.

Concerning the background data, the authors compiled the inventory of the production of electricity power, and disposal by dismantling and pyrolysis, using the ecoinvent Library [29], which is a major LCA database including the materials, energy, transport, manufacturing, use, and disposal phases. In the current study, the authors used the energy and waste disposal process data for LCI. In particular, for the production of electricity, the related calculation should account for the electricity mix [30]. Consequently, the authors generally converted electrical power to electricity generated by thermal, hydro, and nuclear power according to the electricity mix in China [31]. Correspondingly, the metals, printed wire board (PWB), and plastics from the dismantling and crushing steps, and the pyrolysis residue, were disposed of by recycling, incineration, or landfill deposit according to the ecoinvent library methods.

### 2.2. Dismantling and Disposal from Battery Pack to Cell

#### 2.2.1. The Dismantling and Crushing from Battery Pack to Cell

The aim of the dismantling and crushing process is to separate the internal materials of cells from other parts of the vehicle battery. Considering the current conditional limits and small experimental scale of the research, the dismantling and crushing processes were performed manually, which also ensures the integrity of the components obtained from the battery pack. In addition, manual disassembly reflects the current status in China, namely that the degree of automation of recycling companies is low, and the labor force is sufficient and cheap. The dismantling process starts with the disassembly of the entire battery pack. The process of dismantling can be divided into three levels: pack, module, and cells, as described in the following:

- Firstly, the process of disassembling battery packs includes an appearance assessment to determine the approximate situation of the battery, removal of the cover, disconnection of the module plug-in, separation of battery modules, removal of the remaining components and accessories, and then collecting and sorting for dismantling results.
- Subsequently, the module dismantling procedure includes the removal of the case, circuit board, wires, bushings, busbar, bracket, covers, heat sink, and LFP cells.
- The next step is the crushing of battery cells. This process aims to separate the internal materials from the cell, as listed in Table 2. The first step involves deep discharge by a slide resistor to completely eliminate residual electricity for safe crushing. Then, the cell is crushed, and the shell and separator are removed. The remaining residue (internal materials) are treated in the following process.
- The authors did not manually disassemble the electric components in this study, and the approximate content of the materials in electric components is roughly obtained through the investigation of vehicle battery manufacturers in China. We assumed that electric components consist of steel, plastics, and PWB with masses of 50%, 35%, and 15%, respectively.
- We used a manual method for wire crushing because of the experimental limitations and for the purposes of simplification, although specific equipment can be obtained, such as a copper wire crusher. The authors assumed the contents of copper and plastic in wires as 55% and 45%, respectively.

#### 2.2.2. Results and Disposal Methods for LCI Calculation

After dismantling and other manual processes, the authors classified the dismantling and crushing results as metals, plastics, PWB, and other residues. Then, the authors determined the waste treatment methods according to the characteristics of the results and generic treatment processes, such as incineration for plastics, recycling for metals, and landfill for glass [32], which can be implemented at present in China.

In detail, the iron, aluminum, and copper parts are mainly composed of a single component, such as the metal structure of the battery, cooling plate, and busbar, which are suitable for recycling. Then, the authors disposed of the metal parts by recycling. Given the different types of plastics, the authors selected incineration disposal for mixed waste plastics. Considering the composition of the electronic components, which mainly include steel housing, plastics, and PWB, the authors used mixed methods for the manual dismantling of the components, recycling for metals and PWB, and incineration for plastics. Then, the authors calculated the relative inventory obtained by the foreground data and background library (ecoinvent) according to the quality of each part.

### 2.3. The Cell Residue Disposal

#### 2.3.1. The Pyrolysis of Cell Scraps

When the dismantling and crushing processes were completed, the authors conducted the disposal of the internal materials of the cells. Based on the previously presented BOM of the cells in Table 2, the authors considered the possible emissions from cell residue. The decomposition of LiPF_6_ generates toxic gases HF, POF_3_, and PF_5_. The chemical reaction equations are expressed in Equations (1) and (2). The decomposition of the blinder polyvinylidene difluoride (PVDF) can release HF gas at more than 310 °C. In addition, the solvents have a low flash point (EC, 145 °C; DMC, 18 °C; and EMC, 25 °C) [33,34], which results in spontaneous combustion if safety measures are not implemented.

Consequently, the authors implemented a pyrolysis process to accelerate the decomposition of the internal materials of the cells and added related treatment processes for the emissions noted above to reduce their harm. Pyrolysis has been previously applied to battery treatment. Espinosa et al. eliminated the moisture in the load and removed the organic material in a battery by heating [35]. Accurec (an environmental technology company that recycles Ni-Cd and Li-Ion batteries from bicycles, EVs, and BEVs; its sorting method mainly uses manual classification) melts spent batteries at suitable temperatures in a resistance heated retort furnace to deactivate the battery according to the melting characteristics of the volatile organic solvents [19]. Umicore sets a pre-heating temperature to evaporate the electrolytes in batteries and reduce the risk of explosions in the furnace [36]. This process is based on the decomposition temperatures of LiPF_6_ (70–90 °C) and PVDF (379 °C), and the boiling points of EC (248 °C), DMC (91 °C), EMC (110 °C)[4].

In this study, the pyrolysis process was implemented using a quartz tube furnace; a tube inlet access to a nitrogen tank, allowing the experiment to be conducted in a nitrogen atmosphere to prevent the combustion of organic solvents before heating; and a tube inlet access to lime solution, which is used to absorb the gas emissions to avoid an environmental impact on the air. The authors set the temperature of the pyrolysis furnace at 400 °C and maintained this for an appropriate time. When no gas was released from the furnace, we regarded the pyrolysis process to be completed.

#### 2.3.2. The Assumptions and Calculations for LCI in the Pyrolysis Phase

Regarding the energy consumption and environmental emissions in pyrolysis, the authors assumed the ideal conditions as follows:

- During the pyrolysis process, the main energy consumption is from electricity power. Firstly, we can record and calculate the average demand value for the disposal of one cell. Then, we can evaluate the electricity consumption for the whole battery pack. Finally, we can convert this to the energy consumption and environmental emissions based on the electricity mix of China [31] and the ecoinvent database.
- The authors assume that organic solvents are completely volatilized into the air. Moreover, the emissions calculation refers to the contents of the BOM of cells.
- The authors assume that all of the element F in the blinder (PVDF) will be transformed into HF gas, and that the fluorine content is 60%. Similarly, the related data can be evaluated based on the principle of mass conservation and the BOM of cells.
- The authors assume that all of the element F in LiPF_6_ will be transformed into HF gas, POF_3_ gas, PF_5_ gas, and LiF solid. Limewater neutralizes the two gases as shown in Equations (4) and (5), and the LiF will be included in the solid residue as shown in Equation (1). Moreover, related data can be calculated in a similar method to that shown previously.
- Regarding the consumption of nitrogen gas, the authors filled the tube with nitrogen gas before heating. Consequently, the authors were able to evaluate nitrogen consumption according to the processing capacity of the heating tube and the number of times pyrolysis was undertaken for a battery pack.

The authors recorded the consumption of materials and electricity, and the amount of solid waste. Then, the LCI data were derived for electricity production according to China’s production mix, and the landfill used for solid waste based on the ecoinvent library. Finally, the authors integrated the overall LCI results during the LFP battery disposal phase from both the background database and the foreground data, which include energy consumption, raw material consumption, and emissions to air, water, and soil.

## 3. Results and Discussion

### 3.1. Foreground Data of LFP Battery Disposal

After dismantling the battery pack and module, and crushing the cells, we collected and sorted the results. These results indicate that one battery pack yields 26.4 kg Fe, 10.59 kg Cu, 12.53 kg Al, 26.6 kg plastics, and 0.71 kg PWB. In particular, the steel/iron primarily comes from the cover plate, screws, brackets, shells of electric components, etc. The aluminum mainly comes from the cooling plate, the cathode foil, and the cover of the cells. The copper parts are from the anode foil and the PVC outer skin wires. Plastics are derived from the case, brackets, separator, and other parts, which can include materials such as PVC and ABS. PWB comes from the electric components, which are the control units in the EV battery. Notably, a deep discharge process was conducted before crushing to ensure safety. The authors utilized a slip resistance in the range of 0 to 20 Ω for discharging to 0 V.

The pyrolysis process took 1 h for two cells on average. Thus, the authors calculate that 230 kWh of electricity is needed based on the rated power of the furnace (2.5 kW). Based on the assumptions and analysis presented in Section 2.3, the authors determined the emissions during the pyrolysis process for all of the cells. According to the BOM of the cell in Table 2, the content of LiPF_6_ in one cell is approximately 20 g. In addition, the authors argue that the fluorine content of PVDF is 59% and decomposes completely during the pyrolysis using the previous assumption, which releases 0.543 kg HF gas for one battery pack. Then, the authors evaluate the amount of precipitate and the demand of limewater (replaced by the demand for CaO in the calculation) for one battery pack according to the Equations (1)–(5): CaF_2_ 5.81 kg, Ca_3_ (PO4)_2_ 3.78 kg, CaO 6.22 kg. Similarly, the authors evaluate the other emissions during the pyrolysis phase: DMC 5.52 kg, EMC 4.6 kg, EC 5.52 kg. In addition, the demanded amount of nitrogen gas according to the number of times pyrolysis was undertaken (92) and the volume of the pyrolysis tube (Φ50 * 1000) is about 180.55 L (0.23 kg). After pyrolysis, the authors measured the residue and found 51.3 kg residual solid waste is obtained, including the LiF. Therefore, the composition of the foreground data is mainly shown in Table 3 below.

### 3.2. The Total LCI of the LFP Battery Disposal Phase

Due to the manual operation, no energy consumption or emissions result from the process of dismantling and crushing. Consequently, the authors need to calculate the LCI during the metal, plastic, and PWB disposal processes, including recycling or incineration, using the ecoinvent library, and the landfill processing of pyrolysis residue. These results are then connected with the Chinese electricity mix, as shown in Table 4 [31]. Table 4 indicates the main sources for electricity production in China 2017, which are thermal power (71.0%), hydropower (18.6%), wind power (4.7%), and nuclear power (3.9%). In particular, coal plays a dominant role in thermal power, with a contribution of 91.9%. Consequently, the authors allocate electricity consumption to the four sources as 163.29, 42.76, 10.87, and 8.89 kWh, respectively, and assume that thermal power is completely fueled by coal. Finally, the energy consumption and environmental emissions of electricity production are calculated using the ecoinvent library.

Finally, the authors achieve the total LCI of the LFP battery disposal phase by integrating the foreground and background data. The results shown in Table 5 indicate that:

- Regarding energy and material consumption, the majority of hard coal is mainly used in the metals and PWB recycling processes (49.6%), and electricity production (50.2%). Brown coal, crude oil, natural gas, calcite, and clay are mainly consumed in the metals and PWB recycling processes (94.9%, 89%, 90.7%, 93%, and 91.2%, respectively). Oxygen and calcium oxide consumption is mainly in the incineration of plastics (97.9%). Calcium oxide consumption is from the pyrolysis process. The nitrogen is mainly consumed in the metals and PWB recycling processes (53%) and pyrolysis (46.9%) process. Gravel is used in the metals and PWB recycling processes (59.8%), landfill of pyrolysis residue (22.8%), and electricity production (15.2%).
- Regarding emissions to the air, the majority of carbon dioxide emissions come from the metal and PWB recycling processes (48.8%), the incineration of plastics process (14.8%), and electricity production (34.8%). Carbon monoxide and noble radioactive gas come mainly from the metal and PWB recycling processes (87.5% and 82.4%, respectively). Heat waste is mainly from the incineration of plastics process (99.4%), PM2.5/PM10 comes from the metal and PWB recycling processes, and solvents come from the pyrolysis process. Methane and sulfur dioxide are produced mainly by the metals and PWB recycling processes (63.9% and 69.5%, respectively), and electricity production (36.7% and 30.1%, respectively). Nitrogen gas emissions are produced mainly by each of the processes, with the exception of the incineration of plastics process.
- Regarding emissions to water and soil, the majority of emissions of dissolved organic carbon (DOC), total organic carbon (TOC), and chemical oxygen demand (COD) come from the landfill treatment of pyrolysis residue, accounting for 89.2%, 89.2%, and 70.5%, respectively. The majority of emissions of biological oxygen demand (BOD5) mainly come from the metals and PWB recycling processes (26.3%), and the landfill treatment of pyrolysis residue (70.1%). The majority of the emissions of chloride come from the metal and PWB recycling processes (48.2%), and electricity production (35.9%). Heat waste is mostly from the incineration of plastics (99.5%). Emissions of sulfate, sodium, magnesium, and hydrogen-3 are mainly caused by the metals and PWB recycling processes (51.2%, 73.8%, 52.5%, and 61%, respectively), and electricity production (46.7%, 19.1%, 48.1% and 37.9%, respectively). Cadmium emissions are due mainly to the metal and PWB recycling processes (76.5%), and the landfill treatment of pyrolysis residue (20.9%). The emissions to soil comprise calcium phosphate and calcium fluoride, which come from the pyrolysis process.

Due to the low recycling value of LFP batteries, there are relatively few recycling studies on them, especially those involving the cell treatment. At the same time, many previous LCA studies of vehicle batteries have focused on the analysis and research of LCIA [37,38,39,40]. However, there are less data displayed of LCI, which leads to a gap between the research in this article and the existing research results for direct comparison. In this study, the authors make the comparison on LCI in the recycling phase between the LFP battery and the NCM battery.

Table 6 shows part of the inventory data comparison between LFP recycling in this study and an NCM battery recycled by the hydrometallurgy method [41]. The results show that NCM battery recycling requires more types of materials than LFP battery recycling. This is because the hydrometallurgy process is more complicated and requires more materials. On the other hand, compared to the treatment of LFP batteries, the recovery rate and value of hydrometallurgy process are also much higher.

The power requirements of the two methods are relatively close. The recycling of LFP batteries produces higher emissions than NCM batteries, especially carbon dioxide emissions. This is because of the electricity mix difference between China and the USA, as China’s electricity production largely comes from the consumption of fossil energy, which results in a large amount of greenhouse gas emissions.

### 3.3. The Industrial Scale Outlook

It is estimated that the number of waste lithium-ion batteries will be 11.36 million in China in 2030 based on their calendar lifespan [42]. Therefore, the application of battery recycling industrialization will be coming soon. The scale in this study is the experimental level for LFP battery disposal, whereas the industrial scale is the final goal for an LCA/LCI study. In particular, a number of differences exist between the laboratory and industrial scales in LCA/LCI results. Kralisch and Kreisel [43] analyzed the synthesis of m-anisaldehyde, and the results indicate that the advantages consist of savings in energy consumption, the reduction in solvents, and the increase in the reaction yield at the micro-scale. Furthermore, the avoidance of a cryogenic system by increasing the reaction temperature is the most important feature at the industrial scale. However, it can be a challenge to obtain detailed industry data. Consequently, in a follow-up study, we may scale up the weights of known models (Lombardi 2003) [44] or make use of literature-based data supplemented with discussions from industry, rather than rely on a primary data source (Brentner et al. 2011) [45].

By applying the industrial scale process, the consumption of energy and materials, and the quantity of emissions, reduced, and the efficiency of battery disposal can be improved due to economies of scale. In particular, automatic equipment can be adopted for dismantling batteries due to the development of standardized battery design and production. By using a dedicated discharge device, batteries can be discharged at the module or pack stages; thus, significantly shortening the total time required. The cell crushing process can be implemented using a continuous feeding crusher, which can also control components of the atmosphere, such as temperature and gas protection. In addition, wire crushing can also be achieved using a copper wire crusher, which is considerably more efficient than the manual process. Gases produced by pyrolysis can be passed through a scrubbing column, rather than sending them directly into limewater; thus, absorbing them into the liquid of the process water and avoiding leakage during gas treatment. In addition, hydrated lime can be added to the process water used to absorb the gases, and process water can be reused in the scrubbing column after the precipitates are filtered.

In addition, according to the total LCI results above, the metal and PWB recycling processes, and electricity production, play a dominant role in energy and material consumption, and production of emissions. We can presume that recycling equipment also has a high demand for electricity. Consequently, the electricity mix is a highly significant factor for emission results. In the future, when LFP disposal is conducted at the industry level, additional direct data can be obtained, and an LCI effect contribution analysis can be conducted on the changes in the electricity mix from the perspective of sustainable development.

### 3.4. Research Limitation

In this study, the authors discuss the dismantling of spent LFP batteries and the methods of cell disposal and their life cycle inventory. In fact, after the vehicle batteries are retired from electric vehicles, they often have the potential value of cascading utilization. Therefore, further LCA/LCI research will discuss the cascade use of EV batteries before they are scrapped.

On the other hand, in addition to landfilling, the disposal of pyrolysis products needs to discuss whether there are other recycling methods, such as used as industrial fillers, or other methods to recover the lithium element, which are also a valuable research direction.

In addition, with China’s increasing attention to sustainable energy and clean energy, the ratio of renewable energy in China’s electricity mix will increase, while the ratio of fossil energy will decrease. Therefore, future LCA/LCI analysis should consider the impact of these influences.

## 4. Conclusions and Recommendation

During the manual dismantling of the LiFePO_4_ EV battery pack and module, and the crushing of its cells, the authors obtained electronic components, plastics, and several metals, including iron, copper, and aluminum, despite the lack of precise metals in the cells. The components resulting from the dismantling and crushing processes can be disposed of using different methods, such as recycling and incineration. Regarding the cells, the authors conducted the discharge and pyrolysis process for the safety treatment of the internal materials, and then used neutralization and landfill treatment for the waste gases and solid residue, respectively. Using the background database and the foreground data obtained from the analysis and measurement conducted during the LFP battery disposal, the authors compiled a detailed LCI for the waste treatment scenario, which included the consumption of energy and materials, and the emissions to air, water, and soil. In addition, the results show the contribution of each phase to the overall LCI. We can conclude that the inventory can provide the basis for future LCA of LFP batteries.

By comparing the LCI of LFP recycling with that of the NCM battery recycling, the results show that NCM battery recycling requires more types of materials than LFP battery recycling. This is because the hydrometallurgy process is more complicated and requires more materials. On the other hand, compared to the treatment of LFP batteries, the recovery rate and value of the hydrometallurgy process are also much higher. Meanwhile, the recycling of LFP batteries produces higher emissions than NCM batteries, especially carbon dioxide emissions. This is because China’s electricity production mainly relies on fossil fuels, which results in a large amount of greenhouse gas emissions. Consequently, future research on the LCA/LCI of vehicle batteries should take into account the changes in electricity mix.

Subsequent research can integrate the energy consumption and environmental emissions data of raw material extraction, material production, manufacturing and assembly, transport, and use phases into the LCA of LiFePO_4_ vehicle batteries. In addition, research and development of the process of spent LiFePO_4_ battery disposal at the industrial scale is also an important issue in the context of the greater responsibility for both environmental and economic benefits.

## Figures and Tables

**Table 1:** The technical parameters of battery pack and cell.

**Battery Pack**	Weight	150 kg
	Capacity	11.8 kwh
	Rated voltage	300 V
	The number of modules	4
	The number of cells	184
**Cell**	Weight	500 g
	Capacity	20 Ah
	Nominal voltage	3.3 V

**Table 2:** The bill of materials of a LiFePO_4_ cell.

**Materials**	LiFePO_4_	Graphite	Copper foil	Aluminum foil	Aluminum film	Separator
**Mass%**	35	17	10.5	5	3.5	5
**Materials**	DMC (dimethyl carbonate)	EMC (ethyl methyl carbonate)	EC (ethylene carbonate)	LiPF_6_	carbon black	PVDF
**Mass%**	6	5	6	4	2	1

**Table 3:** The foreground data during the LiFePO_4_ (LFP) battery disposal.

Energy and Materials Consumption			Materials, to Further Disposal (kg)					Emissions to Air (kg)			Emissions to Solid (kg)		
Electricity (kWh)	CaO (kg)	Nitrogen Gas (L)	Iron	Aluminum	Copper	PWB	Plastics	DMC	EMC	EC	Ca_3_(PO_4_)_2_	CaF_2_	Solid Waste
230	6.22	180.55	26.4	12.53	10.59	0.71	26.6	5.52	4.6	5.52	3.78	5.81	51.3

**Table 4:** The electricity mix in China 2017.

Electricity Mix	Quantity (Twh)	Details	
Total Electricity production	64,171	-	-
Thermal power	45,558	By coal	41,498 Twh
		By gas	2028 Twh
		By oil	27 Twh
Hydropower	11,931	Pumped Storage	328 Twh
Wind power	3034	-	-
Nuclear power	2481	-	-
Solar Power	1166	-	-
Others	1	-	-

**Table 5:** The total life cycle inventory (LCI) during the disposal of a spent LiFePO_4_ vehicle battery.

Energy and Materials Consumption, Emissions	Recycling of Metals and PWB	Incineration of Plastics	Pyrolysis	Landfill of Pyrolysis Residue	Electricity by Coal	Electricity	Total	Unit
**Energy and materials**								
Energy from others ^1^	38.52	0.32	-	0.89	19.14	187.35	227.07	MJ
Coal, brown	9.11	-	-	-	0.49	0.49	9.60	kg
Coal, hard	91.59	0.07	-	0.17	92.62	92.70	184.54	kg
Oil, crude	10.01	0.08	-	0.26	0.87	0.89	11.24	kg
Natural gas	11.68	0.24	-	0.07	0.85	0.88	12.87	m^3^
Oxygen	1.53	73.95	-	-	-	-	75.48	kg
Nitrogen	0.26	0.00	0.23	-	-	-	0.49	kg
Gravel	23.15	0.81	-	8.82	4.68	5.91	38.70	kg
Calcite	7.08	0.52	-	-	0.58	0.77	8.38	kg
Clay	1.14	0.11	-	-	-	-	1.25	kg
Calcium oxide	-	-	6.22	-	-	-	6.22	kg
**Emissions to air**								
Carbon dioxide	260.78	79.27	0.00	8.36	185.97	186.16	534.57	kg
Carbon monoxide	2.03	-	-	-	-	0.29	2.32	kg
Heat, waste	3.79	707.56	-	-	0.27	0.31	711.66	MJ
Methane	1.08	-	-	-	0.62	0.62	1.69	kg
Nitrogen oxides	0.82	-	0.23	1.06	0.34	0.34	2.44	kg
Noble gas, radioactive	695.27	2.82	-	10.88	131.80	134.75	843.72	kBq
PM2.5	0.23	-	-	-	-	-	0.23	kg
PM10	0.15	-	-	-	-	-	0.15	kg
Sulfur dioxide	1.71	-	-	-	0.74	0.74	2.46	kg
DMC	-	-	5.52	-	-	-	5.52	kg
EMC	-	-	4.60	-	-	-	4.60	kg
EC	-	-	5.52	-	-	-	5.52	kg
**Emissions to water**								
BOD5, Biological Oxygen Demand	0.36	0.05	-	0.96	-	-	1.37	kg
COD, Chemical Oxygen Demand	1.51	0.17	-	4.05	-	-	5.74	kg
DOC, Dissolved Organic Carbon	0.36	0.08	-	3.67	-	-	4.11	kg
TOC, Total Organic Carbon	0.37	0.08	-	3.67	-	-	4.11	kg
Chloride	1.73	0.17	-	0.40	1.29	1.29	3.59	kg
Heat, waste	0.85	162.79	-	-	-	0.01	163.64	MJ
Hydrogen-3	63.66	-	-	1.11	12.80	39.53	104.30	kBq
Magnesium	0.82	-	-	-	0.74	0.75	1.56	kg
Sulfate	5.83	0.05	-	0.18	5.31	5.31	11.37	kg
Cadmium	2.67	0.08	-	0.73	-	0.02	3.49	kg
Sodium	2.82	-	-	0.27	0.73	0.73	3.82	kg
**Emissions to soil**								
Calcium phosphate	-	-	3.78	-	-	-	3.78	kg
Calcium fluoride	-	-	5.81	-	-	-	5.81	kg

^1^ “Energy from others” represents the converted energy from the wind, geothermal, solar, potential, and biomass.

**Table 6:** Inventory data for the recycling of 1 kWh waste LFP battery and nickel cobalt manganese lithium (NCM) 622 battery.

Category	LFP Recycling		NCM Recycling		Unit
	Name	Value	Name	Value	—
Materials	Gravel	3.28	H2SO4 (98%)	9.6	kg
	Calcite	0.71	HCl (30%)	0.3	kg
	Clay	0.11	NaOH (30%)	16.3	kg
	Calcium oxide	0.53	Na2CO3	0.2	kg
	—	—	Extracting reagent	17.4	g
	—	—	Kerosene	42.5	g
	—	—	H2O2	3.2	kg
	—	—	Industrial water	121.6	kg
	—	—	Li2CO3	1.1	kg
Energy	Electricity	15.87	Electricity	20.3	kWh
	—	—	Natural gas	1.2	m3
Emission	Sulfur dioxide	0.21	Sulfur dioxide	0.01	kg
	Carbon dioxide	45.3	Carbon dioxide	0.6	kg
	Carbon monoxide	0.19	Dust	3.1	kg
	Nitrogen oxides	0.21	—	—	kg
	PM2.5	0.019	—	—	kg
	PM10	0.013	—	—	kg
